# Temperature and land use influence tree swallow individual health

**DOI:** 10.1093/conphys/coab084

**Published:** 2021-10-25

**Authors:** Joseph Corra, S Mažeika P Sullivan

**Affiliations:** 1 Office of Research and Development, U.S. Environmental Protection Agency, 26 Martin Luther King Dr., Cincinnati, OH 45268, USA; 2 School of Environment and Natural Resources, The Ohio State University, 125 Heffner Bldg 352 W. Dodridge St., Columbus, OH 43202, USA

## Abstract

Aerial insectivorous bird populations have declined precipitously in both North America and Europe. We assessed the effects of insect prey availability, climate and shifts in water quality associated with urbanization on haematocrit, haemoglobin concentration and heterophil–lymphocyte (H/L) ratios among ~13-day-old tree swallow (*Tachycineta bicolor*) nestlings in the Columbus, Ohio area. Higher mean temperature and increased frequency of extreme heat days during the early breeding period (May–June) were linked to reduced nestling physiological condition as evidenced by lower concentrations of haemoglobin and haematocrit, potentially due to increased heat stress, shifts in insect prey availability or altered parental provisioning efforts. Urbanization and the size and density of emergent aquatic insects were associated with elevated physiological stress, whereas higher mean temperatures and terrestrial insect size were related to lower stress as measured by H/L ratios. Overall, these findings highlight the complex environmental conditions driving nestling health, which may be indicative of post-fledging survival and, consequently, population growth. Our results underscore the need for conservation approaches that adequately address the interrelated effects of changes in climate, land use and food resources on aerial insectivorous birds.

## Teaser

We investigated the influence of environmental conditions on tree swallow nestlings by using haematological measures. Extreme heat was linked to diminished physiological condition. Stress levels were related to urbanization, insect prey availability and temperature. We show that complex conditions drive nestling health on breeding grounds.

## Introduction

Bird populations have experienced considerable declines in North America since the mid-20th century (Rosenberg *et al.*, 2019). Population losses are especially pronounced among aerial insectivores, a guild comprising swallows, swifts, flycatchers and nightjars, in both North America (Nebel *et al.*, 2010) and Europe (Bowler *et al.*, 2019). Multiple drivers are likely contributing to these population declines (Norris *et al.*, 2021; Sullivan *et al.*, 2021). The observed decline in North American aerial insectivorous birds intensifies along a northeastward gradient (Nebel *et al.*, 2010), implicating climate change as one driver of population shifts (Cox *et al.*, 2019).

Recent evidence also has pointed to decreases in the global abundance and biomass of insects (Hallmann *et al.*, 2017), which in part could also be linked to climate change (e.g. Lister and Garcia, 2018). Parallel declines in insects and aerial insectivore populations suggest a link between these phenomena (Møller, 2019). However, the patterns of insect decline are not uniform globally (van Klink *et al.*, 2020), complicating this assumption. Rather than overall abundance or biomass, shifts in the quality of insect prey may be a driver of population declines among aerial insectivores (Spiller and Dettmers, 2019). Recent work by Twining *et al*. (2016, 2018) provides strong evidence that aquatic insects provide critical nutritional benefits to aerial insectivorous birds; accordingly, other studies of aerial insectivore diets have demonstrated a reliance on aquatic insect subsidies (e.g. Beck *et al.*, 2014; Michelson *et al.*, 2018).

Riparian zones are important nesting and foraging sites for insectivorous birds, which are trophically linked to aquatic environments via emergent aquatic insect prey (Nakano and Murakami, 2001; Baxter *et al.*, 2005) For instance, Kautza *et al.* (2016) showed that riparian-nesting swallows derived 41% of their energy from aquatic primary production via emergent aquatic insects. Further, continent-wide analysis by Manning and Sullivan (2021) showed that some aerial insectivorous bird species responded strongly to the abundance of pollution-sensitive emergent insect taxa.

Urbanization has intense effects on bird abundance and community structure (Blair, 1996; Marzluff, 2001), and the consequences of urbanization on riparian birds may be especially pronounced (Rottenborn, 1999). Insectivores may be especially susceptible to the impacts of urbanization due to the associated shifts in food resources (Teglhøj, 2017). Urbanization of watersheds is associated with alteration of riparian food webs due to shifts in aquatic insects (Jones and Clark, 1987; Allan, 2004; Kautza and Sullivan, 2014) and can thereby affect terrestrial insectivores that rely on aquatic insect subsidies (Alberts *et al.*, 2013). For example, Rodewald *et al.* (2013) revealed that the aerial insectivorous Acadian flycatcher (*Empidonax virescens*) fledged fewer young in riparian zones along an urbanization gradient. Riparian urban-breeding birds with a high reliance on emergent aquatic insects may also suffer from higher contaminant loads (Alberts *et al.*, 2013; Sullivan *et al.*, 2021). Overall, detrimental impacts to nestling development, physiological condition and nest survival have been identified among riparian-nesting birds relative to gradients of urbanization (e.g. Morrissey *et al.*, 2014; Becker and Weisberg, 2015). Conditions during the nesting period may have long-ranging implications. The physiological condition and health of nestlings have been identified as predictors of both fledging success (Markowski *et al.*, 2015; Lamb *et al.*, 2016) and post-fledging survival (Vitz and Rodewald, 2011; Naef-Daenzer and Grüebler, 2016; Evans *et al.*, 2020), two metrics identified by Cox *et al*. (2018) as key determinants of population growth among aerial insectivorous tree swallows (*Tachycineta bicolor*).

Haematological parameters can serve as robust indicators of avian health and condition (Booth and Elliott, 2002). For instance, haematocrit (i.e. the proportion of red blood cells) and haemoglobin concentration have been related to other measures of condition such as body fat stores (Minias, 2015) and body mass (Lill, 2011). Increased availability of arthropod prey has been linked to higher haemoglobin concentration (Banbura *et al.*, 2007). For instance, Merino and Potti (1998) showed that experimental diet supplementation in the aerial insectivorous pied flycatcher (*Ficedula hypoleuca*) resulted in increased haematocrit. Further, higher values of haemoglobin concentration and haematocrit have been associated with increased fledging success (Kalinski *et al.*, 2012; Markowski *et al.*, 2015). Leukocyte profiles, derived from complete blood counts (CBCs), are also used as diagnostics of health and physiological stress (Shutler *et al.*, 2004). In particular, the ratio of heterophils to leukocytes (hereafter, H/L ratio) is commonly used to evaluate stress response in wild birds (Davis *et al.*, 2008). Ratios of leukocytes are indicative of stress related to nutritional status (Gross and Siegel, 1983) and may thereby be linked to food availability and other environmental factors (Pigeon *et al.*, 2013; Powell *et al.*, 2013; Wilcoxen *et al.*, 2015). As H/L ratios have been linked to nestling growth (Moreno *et al.*, 2002) and individual recruitment (Lobato *et al.*, 2004), shifts in H/L ratios have potential population-level implications.

We investigated the effect of insect prey availability and quality, urbanization and climate on the physiological condition (i.e. haematological parameters) of nestlings of the riparian-breeding aerial insectivorous tree swallow in greater Columbus, OH, USA. We anticipated that nestling haematocrit and haemoglobin concentration would be positively associated with the following: (i) the availability of insect prey, i.e. the density of insects during the breeding season; and (ii) the quality of insect prey, i.e. both the availability of emergent aquatic insects to provide essential nutrients (Twining *et al.*, 2016) and the average insect body size, given evidence for preferential selection of large-bodied prey among insectivorous birds including tree swallows (Quinney and Ankney, 1985; McCarty and Winkler, 1999; Buchanan *et al.*, 2006). Similarly, we predicted that heterophil-lymphocyte ratios would be negatively related to these metrics. Further, we expected that higher temperatures would be related to increased haematocrit and haemoglobin concentration; Ardia (2013) demonstrated experimentally that increased nest temperature is positively related to nestling haematocrit among tree swallows, possibly due to lower plasma volume. As haemoglobin concentration is typically correlated with haematocrit (Velguth *et al.*, 2010), it could be expected to decline in parallel.

## Materials and methods

### Experimental design

We selected the tree swallow as a model aerial insectivore, as this cavity-nesting species readily nests in artificial nest boxes, facilitating blood sample collection from nestlings (Jones, 2003). Nestlings, rather than adult birds, were selected since haematological parameters may vary by both age and breeding status (Kilgas *et al.*, 2006; Fair *et al.*, 2007; Milenkaya *et al.*, 2013). Nest boxes were constructed according to *Golondrinas de las Americas* (2011), mounted on a combination of steel rebar and electrical conduit to deter predators and deployed in late March each year (2017–2018) at seven riparian study sites in the Scioto River system (OH, USA) distributed across undeveloped and urban land uses (Appendix A, Fig. A1). Each study site consisted of a 500-m river reach based on tree swallow foraging range (Quinney and Ankney, 1985). We deployed 5–6 nest boxes at each site (36 total), approximately in the centre of each 500-m river reach, with ~20 m between boxes to avoid territorial overlap (Muldal *et al.*, 1985). We drew blood samples from the jugular vein (e.g. Sullivan and Vierling, 2012) of two to three nestlings from each brood at age ~13 days for three haematological measures. For each measurement, two to three nestlings from each brood were sampled to provide internal (i.e. nest level) replication and to avoid drawing an excessive volume of blood from any individual bird.

### Haematological parameters

A droplet of blood was collected on a plastic cuvette and placed inside a handheld HemoCue HB 201+ Analyzer (Brea, CA, USA) haemoglobinometer to measure haemoglobin (Hb) concentration in g dl^−1^. To assess haematocrit, a droplet of blood was collected in two glass heparinized microhaematocrit capillary tubes and spun in a portable Hemocue StatSpin centrifuge (Brea, CA, USA). The mean of the resulting packed cell volume (PCV) percentages was computed for each nestling. The H/L ratio was derived from the CBC. A total of 10 μl of blood was collected in an EDTA-coated tube and shipped in an insulated container to Michigan State University’s Veterinary Diagnostic Laboratory (Lansing, MI, USA) for CBC analysis, accompanied by a blood smear (~0.5 μl of blood) prepared in the field on a 25.4 × 6.22 × 1–1.2 mm glass microscope slide.

All work was performed under University, state and federal protocols and permits, including Ohio Division of Wildlife Wild Animal Permit 18-91 and IACUC permits 2011A00000049 and 2009A0215.

### Temperature

We measured temperatures with deployable Thermocron™ Ibutton (Baulkham Hills, NSW, Australia) passive temperature sensors. One sensor was installed inside each nest box at nest box deployment, set to record temperature (°C) at 6-h intervals and downloaded after the breeding season.

### Emergent aquatic and terrestrial flying insects

We sampled insects at each study site for 10 days in mid-late May to align with water chemistry sampling (see below). To adequately capture insect heterogeneity, traps were placed within the 500 m foraging range of tree swallows (Dunn and Hannon, 1992; McCarty and Winkler, 1999) in representative habitats using a methodology consistent with previous studies of riparian arthropods (Rowse *et al.*, 2014; Kautza and Sullivan, 2015; Sullivan *et al.*, 2018). For emergent aquatic insects, we deployed two floating, 1-m^2^ pyramid-style emergent traps at each study site (one trap at each upstream and downstream sections). For terrestrial insects, we deployed two cloth mesh 1 × 1 × 0.6 m Malaise traps (Townes, 1972) in nearshore vegetation at proximal locations upstream and downstream, suspended from trees at a height of ~1 m.

We enumerated and identified invertebrates to family using Johnson and Triplehorn (2005) and Merritt *et al*. (2008), then dried samples in a 60°C oven before weighing. Insects from families with aquatic subtaxa (e.g. Chironomidae) were excluded so that only terrestrial families of flying insects were assessed from Malaise-trap samples. All Hymenoptera were included in the Malaise-trap samples, as relatively few species are aquatic (Bennett, 2008). Non-flying invertebrates and those from taxa thought to be unpalatable to tree swallows (Bellavance *et al.*, 2018) or too small to be a likely food source (McCarty and Winkler, 1999) were also excluded. Flying insect density, measured using the insect capture rate (i.e. no. of insects m^−2^ 10 d^−1^), and mean insect body size (g, dry mass) were calculated from these data.

### Water chemistry

A suite of chemical water-quality variables associated with urbanization in the stream channel (Meyer *et al.*, 2005; Walsh *et al.*, 2005; Vietz *et al.*, 2016) were selected and measured at each study site twice annually (mid-late May and mid-late July, 2014–2018). Nine 250-mg water samples were collected at each study site, one sample each at both stream edges and the thalweg at the upstream, middle and downstream sections each reach. These samples were sent to The Ohio State University’s Service Testing and Research Laboratory (Wooster, OH, USA) for analysis of mercury (Hg; ppt), phosphate (mg l-1), nitrate (mg l-1), ammonia (mg l-1), total phosphorus (mg l-1) and total nitrogen (mg l-1). In addition, water temperature, pH, conductivity and dissolved oxygen (DO) were measured at the same locations with a handheld Hach sensION+ Portable Meter (Loveland, CO, USA). Additional water samples were collected in 60-mg plastic bottles in the thalweg at the upstream, middle and downstream of each reach and analysed in a Hach 2100 N Turbidimeter (Loveland, CO, USA) to measure Nephelometric Turbidity Units (NTUs).

### Land use and land cover

Land cover was quantified using Quantum GIS (QGIS Development Team, 2020) to analyse the data from the 2011 National Land Cover Database (Homer *et al.*, 2011). Land-cover percentage class (developed, forest, and so on) were then computed for a delineated 500-m buffer at each reach. Additional GIS layers were used to calculate the mean human population density (U.S. Census Bureau, 2010), percentage impervious surface (U.S. Geological Survey, 2014a) and percentage canopy cover (U.S. Geological Survey, 2014b) for each 500-m reach buffer.

### Urban stream index

We developed an Urban Stream Index (USI) to capture a gradient of stream urbanization during concurrent work in this study system, via principal component analysis of the water chemistry data and land use and land cover characteristics described above. We derived the USI from PC1, which had an eigenvalue of 5.57 and accounted for 61.9% of the variance, using an approach similar to that of Alberts *et al*. (2013). PC1 was principally influenced by percentage of canopy cover and impervious surface in the 500-m buffer, human population density in the 500-m buffer, DO and Hg concentration in the water column. For further details on the USI methodology, see Sullivan *et al.* (2021).

### Statistical analyses

We performed statistical analyses with the R statistical package version 4.0.2 (R Core Team, 2020) and the R Studio package version 1.2.5042 (RStudio Team, 2020). Distributions of insect body size and capture rate were heavily right-skewed due to outliers, so values were log-transformed before being incorporated into linear-mixed effects models (LMMs). Haemoglobin data were squared and H/L ratios were log-transformed to meet assumptions of normality.

Daily temperature data were used to calculate site-wide temperature means for each site and year, using data collected from May–June (i.e. months in which ~90% of broods hatched). Extreme temperatures were evaluated using methodology employed by Pipoly *et al.* (2013) using a 5-year set of temperature data (2014–2018) collected from the same study sites (see Sullivan *et al.*, 2021). The 90th percentile of all high temperatures recorded May–June was designated the extreme heat threshold, and any day in which the temperature exceeded this threshold (31°C) was counted as an extreme heat day. Days of extreme cold were similarly assessed using the 10th percentile of temperatures, but this measure was not used in statistical models as these data were strongly correlated with mean temperature (Pearson’s correlation: *r* = −0.895, *n* = 198, *P* < 0.001), whereas extreme heat days were only moderately correlated with mean temperature (Pearson’s correlation: *r* = 0.449, *n* = 198, *P* < 0.001).

We developed LMMs using lmerTest version 3.1-2 (Kuznetsova *et al.*, 2017) to test the effects of urbanization, temperature and insect prey on nestling physiological condition (Bolker *et al.*, 2009). Models were fitted with haemoglobin concentration, haematocrit and H/L ratios as response variables. We included insect prey measures (body size, capture rate), temperature variables (mean and no. of extreme heat days), USI and year as fixed effects. Study site and brood (i.e. nest box, the latter nested in study site) were included as random effects. Plots suggested a potential curvilinear relationship between temperature and haemoglobin and haematocrit, so a quadratic term for temperature was included in the global model.

LMMs employed restricted maximum likelihood to facilitate model comparison. For each response metric, we used the R package MuMIn version 1.43.17 (Bartoń, 2020) to generate subsets of a global model containing all predictors and to compute AIC_c_ (Akaike’s information criterion corrected for small sample size), ΔAIC_c_, Akaike weight (ω_i_), *R*^2^ marginal (*R*^2^m) and conditional (*R*^2^c) values. We computed *P*-values of predictors using the R package sjPlot version 2.8.7 (Lüdecke, 2021); *P*-values of <0.05 were used to indicate statistical significance and *P* > 0.05 < 0.10 were used to indicate a trend. Models were ranked by ΔAIC; we retained models with ΔAIC_c_ ≤ 2, (Burnham and Anderson, 2002) that we considered ecologically plausible, excluding models that included no predictors with *P*-values of <0.10 and models where predictor sets were redundant with those of more salient models.

## Results

Among study sites, flying insect density and body size showed only a weak-to-moderate correlation with urbanization, mean temperature or the frequency of extreme heat days (Appendix A, Tables A.1, A.2). Nest box occupancy was 58% across the 2 years of study and ranged from 40% to 80% at each study site in each year. We collected blood samples from 237 nestlings (Appendix A, Table A.3). Three observed outliers among the haematological values (two for haemoglobin and one for H/L ratio) were reviewed and deemed possibly erroneous and were therefore excluded from analyses.

**Figure 1:**
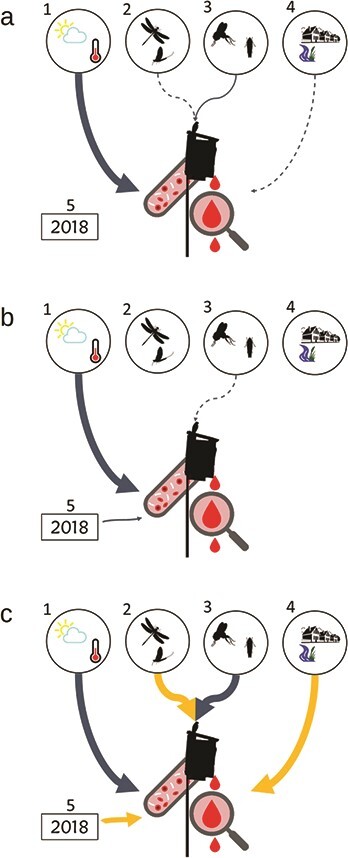
Hypothesized and documented relationships from retained models for tree swallow nestling **(a)** haemoglobin concentration (g dl^−1^), **(b)** haematocrit (% PCV) and **(c)** H/L ratio and **(1)** temperature (°C), **(2)** emergent aquatic flying insect density (no. of insects m^−2^ 10 d^−1^) and body size (g, dry mass), **(3)** terrestrial flying insect density (no. of insects m^−2^ 10 d^−1^) and body size (g, dry mass), **(4)** urbanization (USI) and **(5)** year 2018. Significant relationships (*P* < 0.05) from retained regression models (Table 1) are indicated by thick arrows, trends (*P* > 0.05 < 0.10) by thin arrows and non-significant relationships (*P* > 0.10) by dashed arrows. Gold arrows indicate a positive relationship, while dark grey arrows indicate a negative relationship.

**Figure 2:**
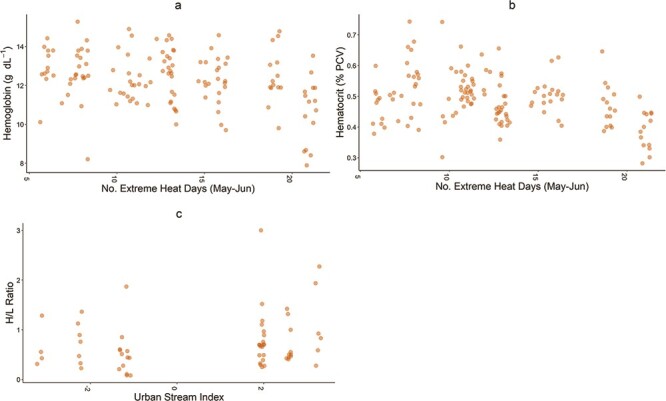
Scatter plots of haematological parameters in tree swallow nestlings (age, ~13 days) and selected significant (*P* < 0.05) predictor variables: (**a**) no. of days of extreme heat (May–June) and haemoglobin concentration (g dl-1), (**b**) no. of days of extreme heat (May–June) and haematocrit (% PCV) and (**c**) the USI and H/L ratio. Points indicate values for individual nestlings.

Temperature variables were prominent in most of our highly supported models (ΔAICc ≤ 2; *p* < 0.05) for haematocrit and haemoglobin concentration (Table 1; Fig. 1a, b). In particular, the frequency of extreme heat days was negatively associated with haematocrit and haemoglobin concentration in nearly all haematocrit and haemoglobin models (*P* < 0.05; Fig. 2a, b). Mean temperature was negatively related to haematocrit values in 50% of the top models in the set (Table 1; Fig. 1b). Our results indicated that the quadratic term for temperature was significant in some models but largely duplicative of models containing extreme heat days as a predictor, so the quadratic term was dropped in favour of the predictive value of extreme heat days.

**Table 1:** Retained regression models (ΔAIC_c_ ≤ 2) with strong ecological rationale for nestling haematological parameters

Model	AIC_c_	ΔAIC_c_	ω_i_	R^2^ (m, c)	df	Emergent insects		Terrestrial insects		Temperature		USI	Year
						Density	Body size	Density	Body size	Mean	No. heat days		
**Haemoglobin** (g dL^−1^)^2^ **(*n* = 134)**	1313	0.00	0.048	0.06, 0.29	5						**−** **0.025**		
	1314	1.04	0.028	0.07, 0.30	6			− 0.281			**−** **0.014**		
	1314	1.08	0.028	0.07, 0.29	6					+ 0.287	**−** **0.011**		
	1315	1.52	0.022	0.06, 0.31	6	− 0.411					**−** **0.026**		
	1315	1.61	0.021	0.03, 0.33	5				**+** **0.093**				
**Haematocrit** (% PCV) **(*n* = 143)**	−352	0.00	0.036	0.16, 0.54	6					**−** **0.045**	**−** **0.038**		
	−351	0.30	0.031	0.18, 0.53	7				− 0.158	**−** **0.051**	**−** **0.011**		
	−351	0.66	0.026	0.15, 0.53	6						− **0.009**		− **0.068**
	−350	1.27	0.019	0.17, 0.52	7				− 0.195		**−** **0.003**		− **0.093**
**H/L ratio** (ln H/L) **(*n* = 61)**	117	0.00	0.397	0.16, 0.54	10	**+** **< 0.001**	**+** **0.001**		**−** **0.017**	**−** **< 0.001**		**+** **< 0.001**	**+** **< 0.001**

Models include AIC_c_ scores, ΔAIC_c_, Akaike weights (ω_i_), marginal (m) and conditional (c) variation explained (*R*^2^) and degrees of freedom (*df*). The symbols ‘+’ and ‘-‘ indicate the direction of the relationship for the variables present in each model, followed by the *P*-value for that variable. Bold text indicates *P*-values that are significant (*P* < 0.05) or evidence of a trend (*P* > 0.05 < 0.10). *n* = number of nestlings from which samples were collected. USI = Urban Stream Index. *R*^2^ m = marginal (i.e. % variation explained by fixed effects); *R*^2^ c = conditional (i.e. % variation explained by random effects). Note that we excluded models that included no predictors with *P* < 0.10 and/or sets of predictors that were redundant with more salient models.

Insect density and body size emerged as salient predictors in five of the nine most highly supported models for haematocrit and haemoglobin. However, few of these relationships were significant (*P* < 0.05); a single model revealed a negative trend between haemoglobin concentration and terrestrial insect body size (*P* = 0.093; Table 1; Fig. 1a). In contrast, the single salient model for H/L ratio showed significant relationships (*P* < 0.05) with both density and body size for emergent aquatic and terrestrial insect prey. However, emergent insect density and body size were positively associated with H/L ratios, while terrestrial insect body size was negatively related to H/L ratios in two models (Table 1). In this same model, mean temperature was negatively associated with H/L ratios (*P* < 0.001). In addition, USI was positively related to H/L ratio (*P* < 0.001; Table 1; Figs 1c, 2c). Across our model sets, random effects explained substantial observed variation: 22–30% for haemoglobin, 35–38% for haematocrit and 38% for H/L ratio (Table 1).

Note that we used site-wide temperature data, with the goal of evaluating both the direct and indirect effects of temperature; i.e. food availability and provisioning ability of parents (e.g. Kalinski *et al.*, 2009). Loss of temperature data occurred in some nests in both years due to instrument failure; as a result, nest-level temperature data was only available for a subset of nest boxes. For broods in these nest boxes, we constructed models using only temperature data collected in that nest box from that brood’s hatch date through age ~13 days (*n* = 83, 87 and 37 for haemoglobin concentration, haematocrit and H/L ratio models, respectively). Temperature predictors were still present though less salient among these models, appearing among the strongest models (ΔAICc, ≤2) less frequently (data not shown).

## Discussion

Our results suggest that local climatic conditions exert moderate to strong influences on tree swallow nestling haematological parameters and, consequently, individual health. Haematocrit and haemoglobin concentration are typically correlated with one another and positively related to body mass in nestling birds (Lill *et al.*, 2013). In the current study, higher temperatures were negatively related to haematocrit and haemoglobin concentration, indicating diminished physiological condition and aligning with other studies that showed detrimental impacts on nestlings due to high air temperatures (e.g. Bourne *et al.*, 2021; Oswald *et al.*, 2021). However, our findings contrast with previous results from the same study system that showed that higher mean temperatures and fewer days of extreme cold were associated with a greater number of fledglings, while the frequency of extreme heat days was related to reduced nestling body mass (Sullivan *et al.*, 2021). Results of the current study also are inconsistent with Ardia (2013), who demonstrated experimentally that higher nest temperatures were related to increased haematocrit among tree swallow nestlings.

The diminished haematocrit and haemoglobin concentration we observed among nestlings may be related to inferior nutritional status. While warmer weather has been associated with improved reproductive output and body condition among insectivorous birds, likely due to increased insect activity at higher temperatures (e.g. Arlettaz *et al.*, 2010; Winkler *et al.*, 2013), extreme heat may be related to inferior trophic conditions. For example, Kalinski *et al.* (2009) linked hot, dry seasonal weather conditions to lower arthropod abundance and, consequently, lower haemoglobin concentration in great tits (*Parus major*), implying that hot weather may drive haematological responses via changes in insect prey availability. However, our data showed only weak or moderate correlations between extreme heat and insects (Appendix A, Table A.2). In addition, extreme heat has been related to changes in provisioning effort by adult birds, with adverse consequences for nestling condition (e.g. Clark, 1987; Wiley and Ridley, 2016). Tapper *et al*. (2020) observed that female tree swallows increased provisioning rates when their ability to dissipate excess heat was experimentally enhanced. Finally, nestling growth, mass and survival may be depressed through direct effects of heat stress (Murphy, 1985; Cunningham *et al.*, 2013). Overall, increasing evidence suggests that chronic exposure to frequent extreme heat conditions has adverse consequences for fitness among birds (Conradie *et al.*, 2019; Andreasson *et al.*, 2020).

Conversely, nestling H/L ratios were negatively related to temperature in the only salient model (Table 1), suggesting that higher temperatures were linked to reduced physiological stress. Further, the salient model for H/L ratios was more complex overall, with size of terrestrial insect prey also related to lower H/L ratios, while emergent insect prey density and size were associated with higher ratios (Table 1). Nutritional status has been linked to H/L ratios (Wilcoxen *et al.*, 2015), suggesting the availability and quality of insect prey may drive these patterns. Twining *et al*. (2016) found that tree swallow nestlings fed diets of aquatic invertebrates rich in long-chain omega-3 polyunsaturated fatty acid grew more rapidly, exhibited greater immunocompetence and had lower basal metabolic rates. In our study system, nestlings exhibited ~38% dietary reliance on emergent aquatic insects (Sullivan *et al.*, 2021). Despite the nutritional benefits conferred by emergent insects, this advantage may be compromised by emergent-insect facilitated biomagnification of contaminants such as mercury (Hg) (Rowse *et al.*, 2014; Twining *et al.*, 2021). Thus, trade-offs between the benefits and risks associated with aquatic insects emerging from polluted aquatic ecosystems may contribute to explaining the negative relationship we observed between emergent insects and physiological stress.

The USI, a metric of urbanization, was also associated with higher H/L ratios (Table 1). Banbura *et al*. (2013) implicated human-induced changes in the structure of breeding habitats and the associated food-related stressors in elevated H/L ratios of nestling blue (*Cyanistes caeruleus*) and great tits. Urban environments have been linked to elevated H/L ratios in some wild birds (e.g. Ruiz *et al.*, 2002), and stressors associated with urban environments like reduced diet quality and light, chemical and noise pollution have been linked to inflammatory responses (Isaksson, 2015) suggesting that urbanization may be a common driver of reduced individual condition. However, given that H:L ratios are known to respond positively to a broad range of stressors (Davis *et al.*, 2008) and may vary among individual birds (Vleck *et al.*, 2000), care must be taken in interpreting these results.

Other than a positive trend between terrestrial insect body size and haemoglobin (Table 1; Fig. 1a), we did not observe significant associations or trends between insect prey availability and haematocrit and haemoglobin concentration, although these relationships have been observed for passerine birds previously (Banbura *et al.*, 2007). Although our emergent insect sampling was rigorous and followed standard protocols (Bellmore and Baxter, 2014), it may not have fully represented preferred prey such as odonates (Mengelkoch *et al.*, 2005). An alternate explanation is that limited variability in aquatic and terrestrial insect community characteristics (e.g. richness, density, body size) among our protected-to-urban study sites precluded significant relationships from emerging.

In all 10 models, *R*^2^c values were substantially higher than *R*^2^m values (Table 1). The influence of random effects (i.e. brood and study site) was considerable for all three response variables in nestling tree swallows, suggesting that other factors—such as parental care—may also be important to consider. Among wild birds, parental health and condition have been related to provisioning effort (e.g. Bowers *et al.*, 2019), with potential implications for nestling haematological parameters. For instance, experimental treatments by Banbura *et al.* (2011) linked improved nutritional status of adult blue tits (*P. major*) to lower H/L ratios among nestlings.

Given links among avian haematological parameters and fledging success (Banbura *et al.*, 2007) and fitness (Bowers *et al.*, 2014), our results underscore the importance of climate as a mechanism for shaping avian individual health and, potentially, demographic shifts (Cox *et al.*, 2020). Our findings also provide evidence that effective conservation paradigms for riparian aerial insectivorous birds will need to include monitoring and management of aquatic and adjacent terrestrial habitats as linked systems. Additional research is needed bridging individual to population consequences of environmental change. Further, because nestling physiological condition has been linked to post-fledging survival (Hylton *et al.*, 2006), our findings highlight the need to assess environmental impacts on nestling health and their role in population dynamics.

## Supplementary Material

AIB_ConPhys_appendix_A_REVISION_Aug2021_coab084
