# Supplementary material for: Temperature and land use influence tree swallow individual health
Source: Conserv Physiol. 2021 Oct 25;9(1):coab084. doi: 10.1093/conphys/coab084 (PMC8546433; doi:10.1093/conphys/coab084)
Supplement: AIB_ConPhys_appendix_A_REVISION_Aug2021_coab084 [file aib_conphys_appendix_a_revision_aug2021_coab084.docx]

**Appendix A.**

**Table A.1.** Mean, standard deviation, median, minimum, and maximum values for emergent aquatic and terrestrial flying insect body size (dry mass, mg) and density (i.e., capture rate, no. of insects m^-2^ 10 d^-1^).

| **Insects** |  | **Emergent** (*n* = 14) | **Terrestrial** (*n* = 14) |
| --- | --- | --- | --- |
| **Body size (dry mass, mg)** |  |  |  |
| Mean (SD) |  | 0.191 (0.117) | 3.590 (3.610) |
| Median [Min, Max] |  | 0.151 [0.049, 0.592] | 2.44 [0.286, 16.800] |
| **Density (capture rate, no. m^-2^ 10 day ^-1^)** |  |  |  |
| Mean (SD) |  | 383 (449) | 74 (116) |
| Median [Min, Max] |  | 168 [19, 1960] | 40 [5, 619] |

**Table A.2.** Pearson’s correlations among Urban Stream Index (USI), mean air temperature (°C), and no. extreme heat days, and emergent aquatic and terrestrial flying insect body size (dry mass, mg) and density (i.e., capture rate, no. of insects m^-2^ 10 d^-1^).

|  | **Emergent** | | **Terrestrial** | |  |
| --- | --- | --- | --- | --- | --- |
|  | **Density** | **Body size** | **Density** | **Body size** | ***n*** |
| **USI** | *r* = -0.200,  *p* = 0.493 | *r* = -0.047, *p* = 0.872 | *r* = -0.496, *p* = 0.072 | *r* = -0.239,  *p* = 0.410 | 14 |
| **Temperature** | *r* = 0.217,  *p* = 0.456 | *r* = -0.093,  *p* = 0.752 | *r* = -0.191, *p* = 0.513 | *r* = -0.444,  *p* = 0.111 | 14 |
| **No. Heat Days** | *r* = 0.107, *p* = 0.728 | *r* = -0.113, *p* = 0.712 | *r* = -0.171, *p* = 0.577 | *r* = -0.485,  *p* = 0.093 | 13 |

**Table A.3.** Mean, standard deviation, minimum, and maximum values for the three hematological parameters for nestling tree swallows (age ~13 d).

| **Hemoglobin** (g dL^-1^) |  |
| --- | --- |
| Mean (SD) | 12.3 (1.5) |
| Median [Min, Max] | 12.5 [6.8, 15.3] |
| **Hematocrit** (% PCV) |  |
| Mean (SD) | 49.2 (8.0) |
| Median [Min, Max] | 49.5 [28.0, 74.5] |
| **H/L Ratio** |  |
| Mean (SD) | 0.77 (0.53) |
| Median [Min, Max] | 0.64 [0.08, 3.00] |


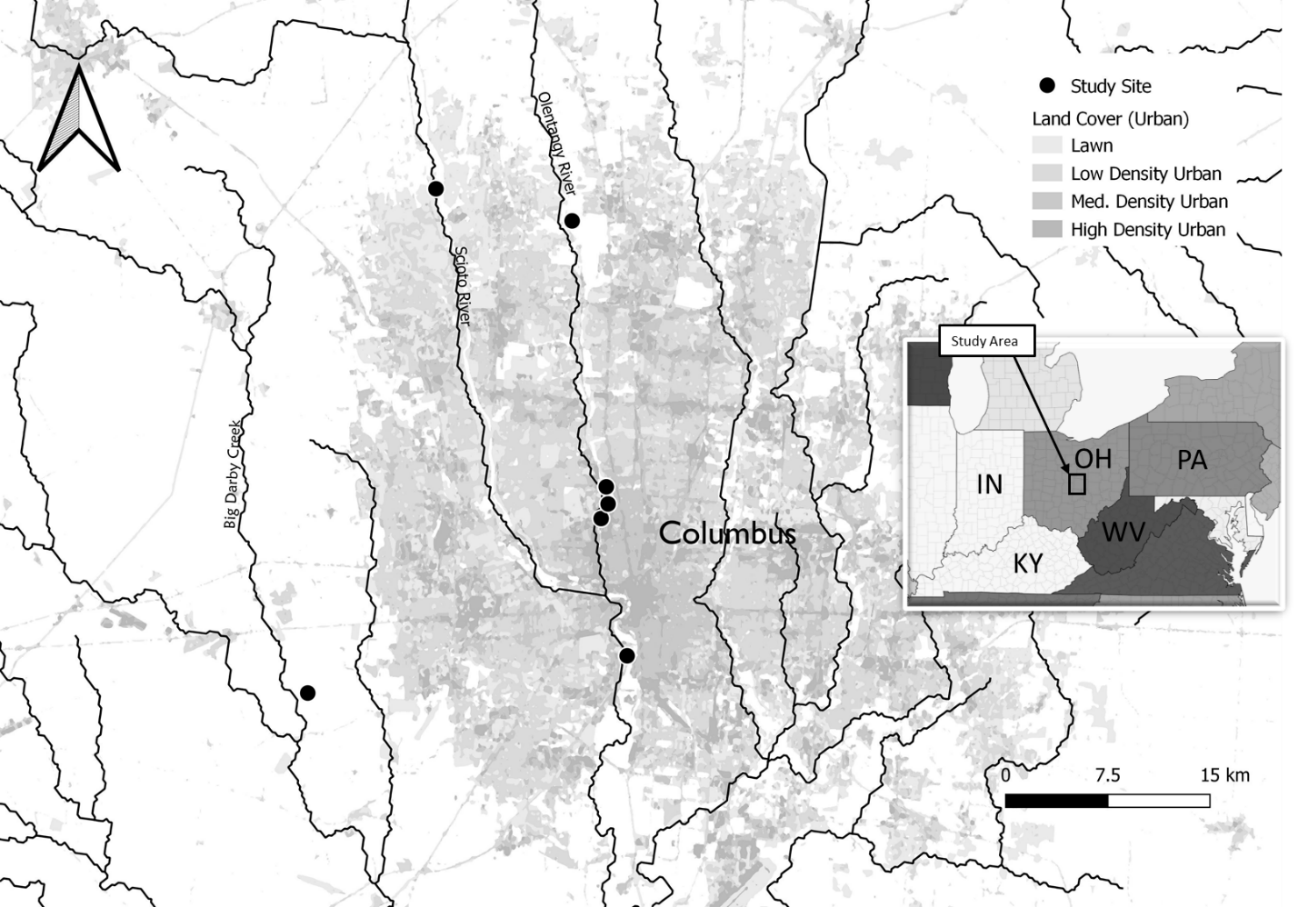


**Figure A.1.** Study sites and urban land cover in the greater Columbus, Ohio area. Inset map shows location of Columbus within larger region. Source: Homer et al. (2011) and QGIS Development Team (2021).


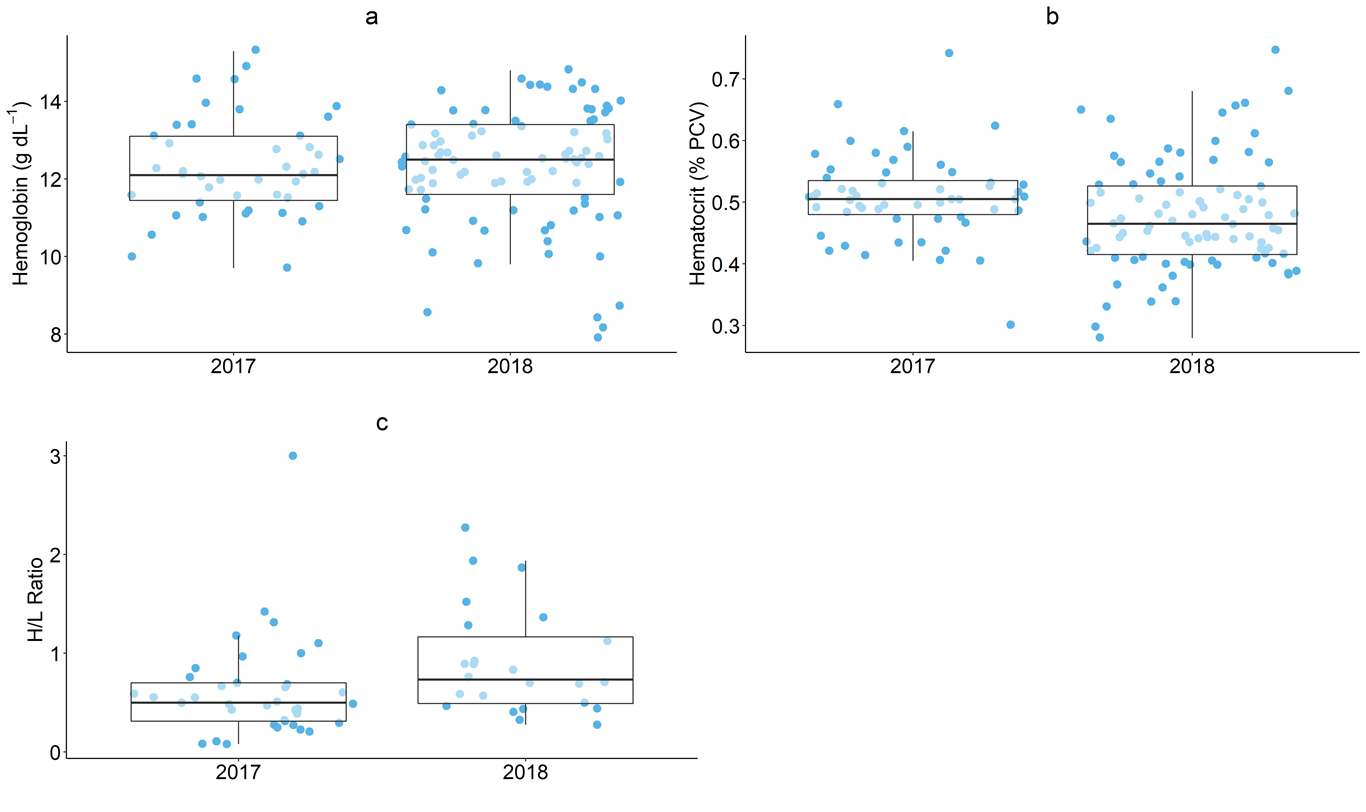
**Figure A.2.** Raw data points overlaid with box-whisker plots (with outlier points removed) for tree swallow nestling **(a)** hemoglobin concentration, **(b)** hematocrit, and **(c)** H/L ratio, by year (2017-18).
